# Internal fixation of delayed union of fracture with chronic osteomyelitis due to Staphylococcus epidermidis: A case report

**DOI:** 10.1016/j.amsu.2020.06.009

**Published:** 2020-06-11

**Authors:** Muhammad Ardi Munir, Pascal Adventra Tandiabang, Prihantono

**Affiliations:** a Department of Social Health Science, Bioethics and Medical Law, Faculty of Medicine, Tadulako University, Palu, 94148, Indonesia; b Department of Orthopedic and Traumatology Surgery, Undata General Hospital, Palu, 94118, Indonesia; c Department of Anatomy, Faculty of Medicine, Tadulako University, Palu, 94148, Indonesia; d Department of Surgery, Faculty of Medicine, Hasanuddin University, Makassar, 90245, Indonesia

**Keywords:** Infection after internal fixation, Staphylococcus epidermidis, Chronic osteomyelitis, Delayed union, Case report

## Abstract

**Introduction:**

The most feared and challenging complication in treating patients with musculoskeletal trauma is infection after fracture fixation, which can delay healing and, in turn, result in permanent functional loss of limbs or even amputation.

**Case presentation:**

Here, we describe a case in which a patient presented with stage 4 chronic osteomyelitis on the right tibia and fibula. To treat the late infection by eradicating or reducing the infection, it was recommended to replace the internal fixation with surgical debridement for 6 weeks and antibiotics for 6–12 weeks until the internal fixation device could be removed.

**Conclusion:**

Delayed union and chronic osteomyelitis are possible complications of IAFF.

## Highlights

• Infection after fracture fixation is the most feared and challenging complication in treating patients with musculoskeletal trauma.
• We report the rare case of a male suffering stage 4 chronic osteomyelitis due to *Staphylococcus epidermidis.*
• Disruption of fracture healing is a clinical symptom that often arises in late infections caused by microorganisms such as *Staphylococcus epidermidis.*

## 1 Introduction

Osteomyelitis, a rare incidence of musculoskeletal infections [1], has existed since ancient times and was first described by Hippocrates [2]. Chronic osteomyelitis is a condition of acute osteomyelitis that has failed to heal. In a patient with chronic osteomyelitis, a few weeks to several months after the onset of acute infections, a sequestrum should be observable via X-ray, and the patient may experience chronic infection and sinus drainage [3]. Although chronic osteomyelitis used to be the dreaded sequel to acute hematogenous osteomyelitis, it now more often follows an open fracture due to trauma or surgery [2].

In developing countries, trauma has become a major public health problem due to increasing industrialization and urbanization. The rise in trauma during the past few decades has prompted an increased incidence of fractures treated with internal fixation [4]. Operative fixation of bone fractures is a highly complex process due to the nature of bone damage, which cannot be predicted preoperatively, and the number of concurrent injuries that can occur should be considered [5].

The most feared and challenging complication in treating patients with musculoskeletal trauma is infection after fracture fixation (IAFF), which can delay healing and, in turn, result in permanent functional loss of limbs or even amputation [5]. IAFF is not only a source of morbidity and mortality but can also add to pre-existing socioeconomic burdens. The incidence rate of successful treatment for IAFF ranges between 70% and 90% [3], and studies have reported an IAFF incidence rate of 1%–2% for closed fractures and 30% with open ones [6].

Here, we describe a case in which a patient presented with stage 4 chronic osteomyelitis on the right tibia and fibula, and reported the case which aligns with SCARE criteria [7].

## 2 Case presentation

**Fig. 1:**
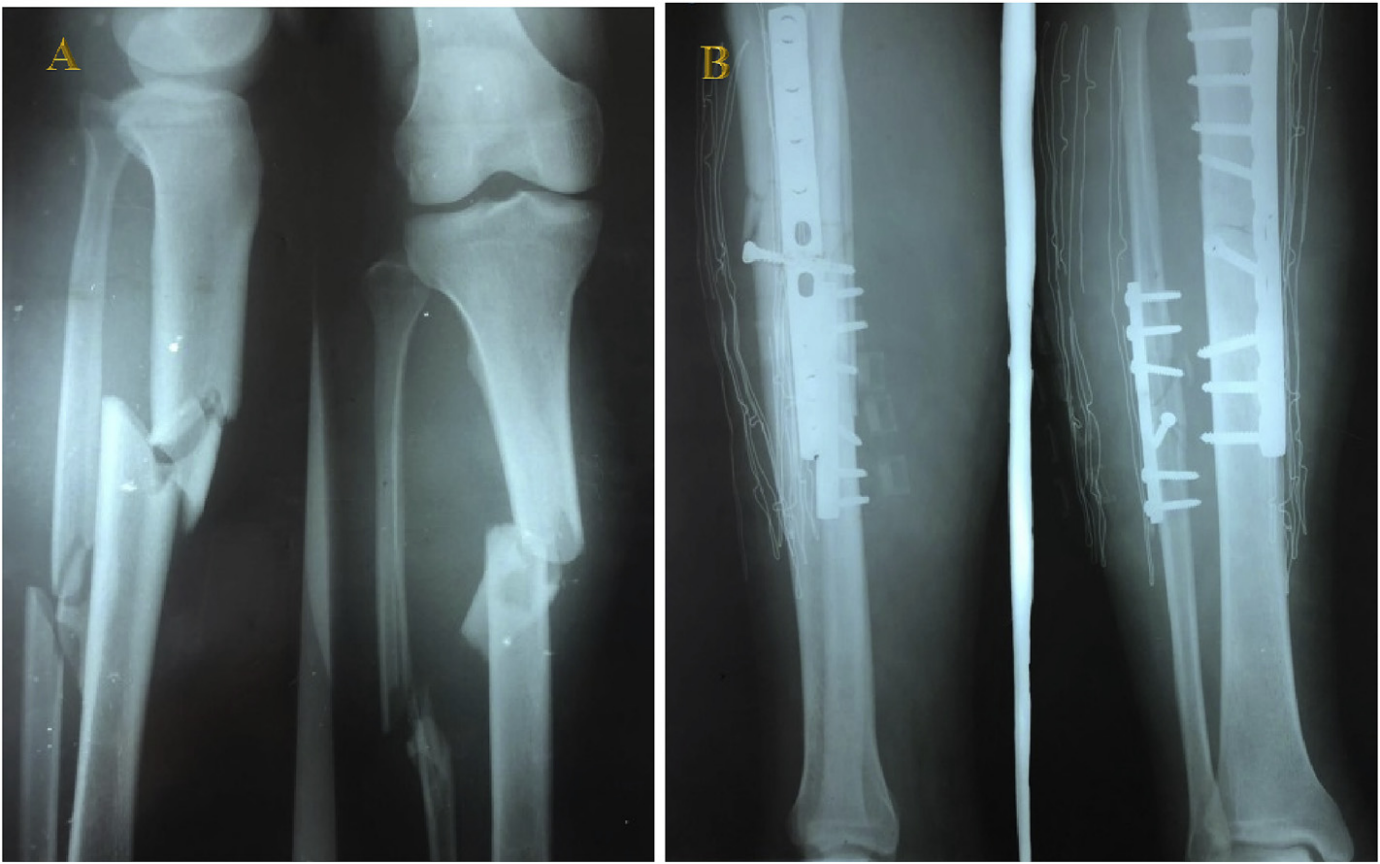
(A) Preoperative AP and lateral X-rays of the right leg showing a fracture of the tibia and fibula. (B) X-ray image of right-leg AP and lateral view showing internal fixation of the fracture.

A 31-year-old male was admitted to our hospital with an open fracture, classified as Gustilo IIIa, of the tibia and fibula on the right leg 2 weeks after a road traffic accident (Fig. 1A). The patient had no comorbidities, no history of musculoskeletal disease, and no notable medical history and reported not smoking or consuming alcohol.

Previously, in a tertiary hospital, the patient underwent debridement and was later referred to our hospital, where we performed open reduction internal fixation (ORIF) surgery (Fig. 1B). The patient had otherwise remained healthy, and the surgical wound healed flawlessly without any active infection in any system. Nevertheless, on Day 2 postoperatively, he received 3 units of blood via transfusion and began mobilization.

**Fig. 2:**
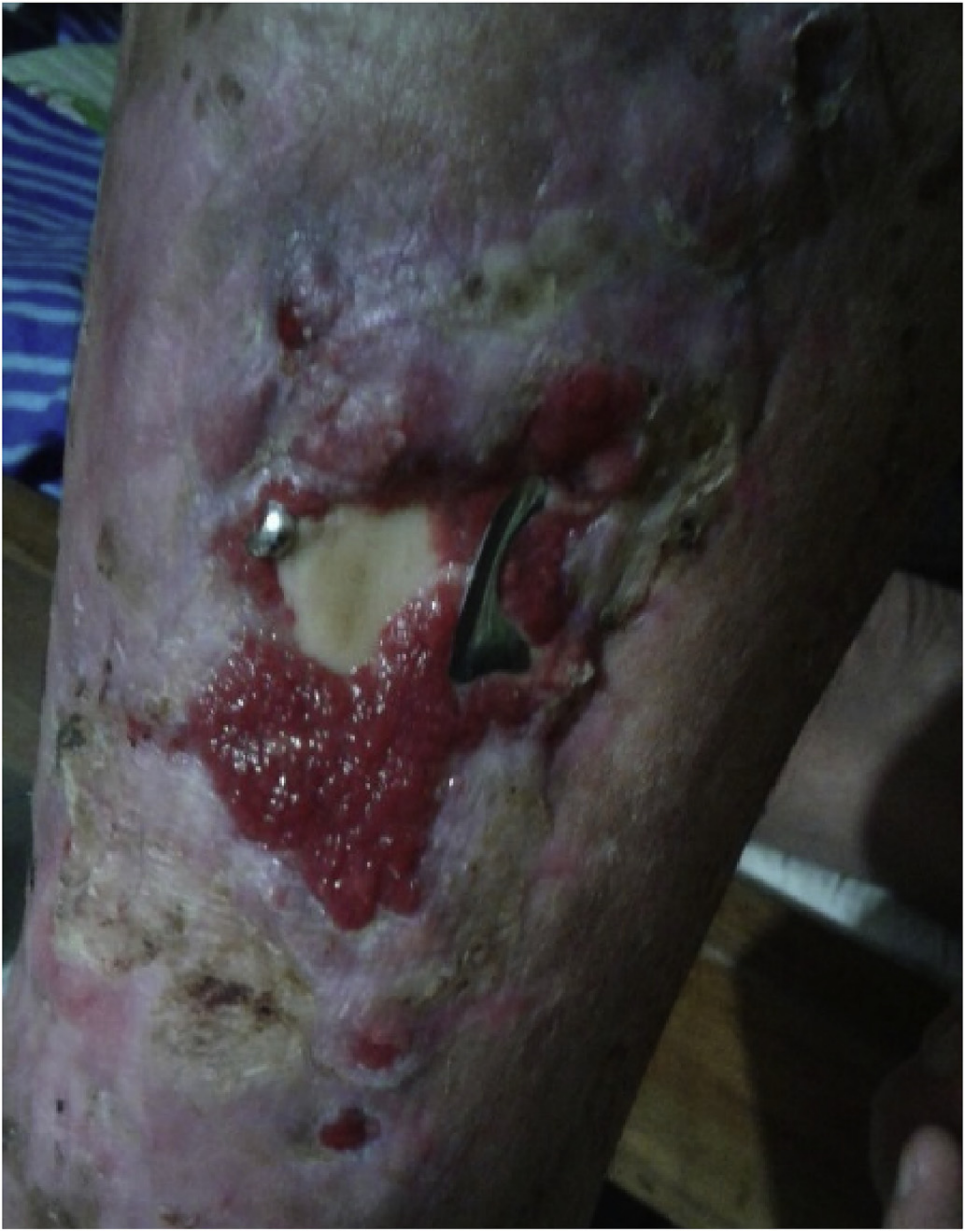
Image of right leg showing an exposed implant.

Upon being admitted to the hospital, the patient was administered 1 g of antibiotic cefuroxime intravenously 3 times per day until receiving none on Day 8 postoperatively. Although discharged to his home 8 days after the operation, he returned 20 weeks after surgery with complaints of implant exposure at the wound site and a fever of 101.3 °F for 4 days. From the outside, the wound appeared tenderness to palpation, although exposed implants measured 2 cm long and 1 cm wide (Fig. 2).

**Fig. 3:**
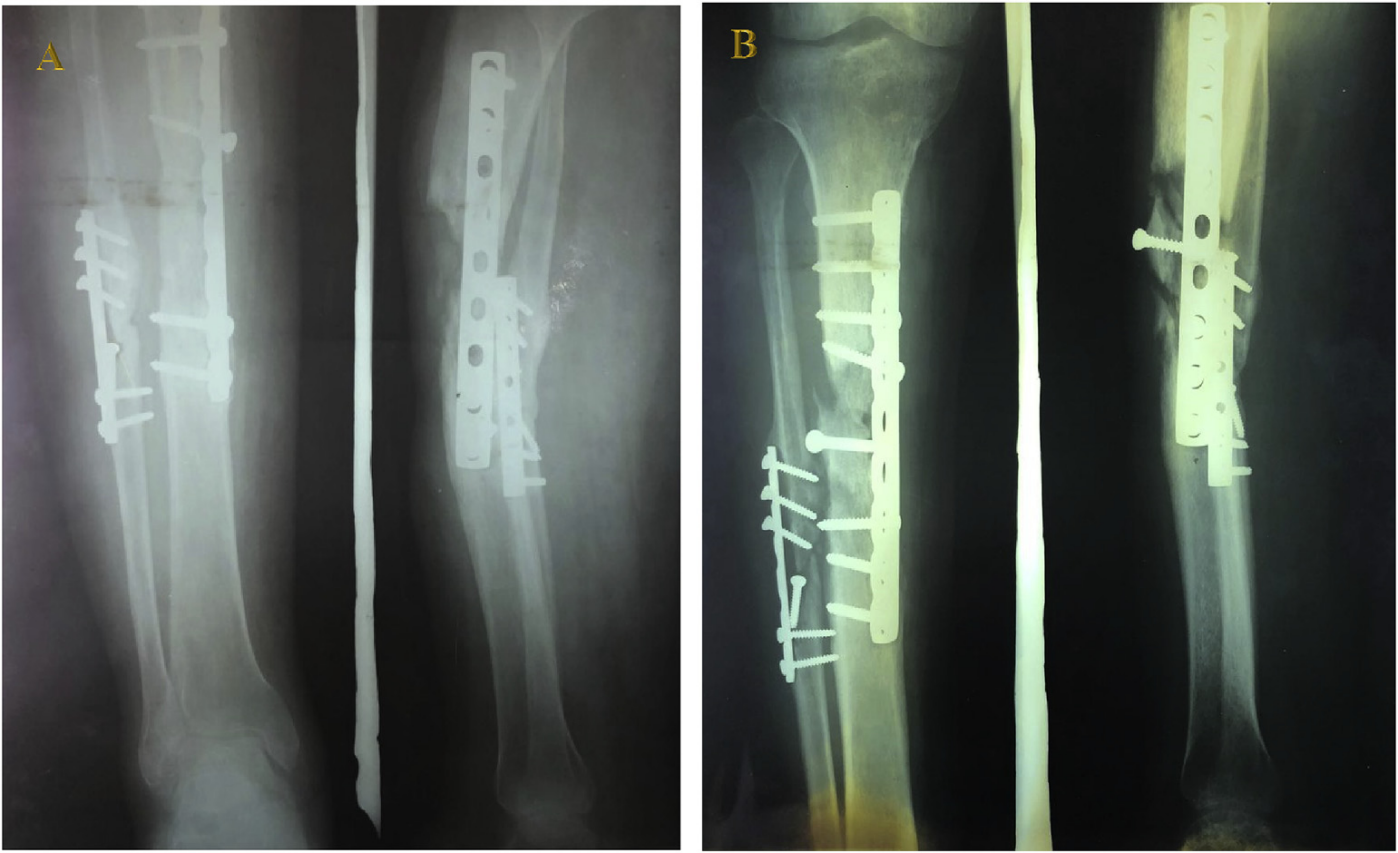
(A) X-ray image of right-leg AP and lateral view showing delayed union and infection of the bone. (B) X-ray image of right-leg AP and lateral view post-debridement and replacement of internal fixation.

The patient underwent radiological examination, and the results showed new bone thickening that formed a sheath, or involucrum, covering the sequestrum and infected tissue (Fig. 3A). Considering the symptoms, physical examination, and radiological examination, the most likely preoperative diagnosis was stage 4 chronic osteomyelitis, according to the Cierny–Mader staging system (Table 1).

**Table 1:** Timeline.

Date	Event	Findings
June 1, 2018	Road traffic accident	Gustilo IIIa open fracture classification of tibia and fibula in the right leg
June 2	Debridement in tertiary hospital	–
June 15	Referred to our hospital	ORIF surgery
June 17	Transfused with 3 units of blood and mobilization began	Anemia
June 22	1 g of antibiotic cefuroxime intravenously 3 times per day	–
June 23	Outpatient	–
November 10	Implant exposure and fever	Clinical and radiologic features showed stage 4 chronic osteomyelitis
November 11	Debridement and replacement of internal fixation	Antibiotic cefazolin intravenously
November 18	Culture results: *Staphylococcus epidermidis* and *Enterococcus faecalis*	Surgical debridement and continuous therapy with cefazolin intravenously
November 25	Outpatient (patient take oral antibiotics for 4 weeks)	

When the elevated temperature persisted, debridement was performed, and when pus was observed, the internal fixation was replaced (Fig. 3B). Cultures taken from bone lesion revealed *Staphylococcus epidermidis* and *Enterococcus faecalis*. Except for *S. epidermidis*, the other infection agents are rarely found in our department and hospitals.

**Fig. 4:**
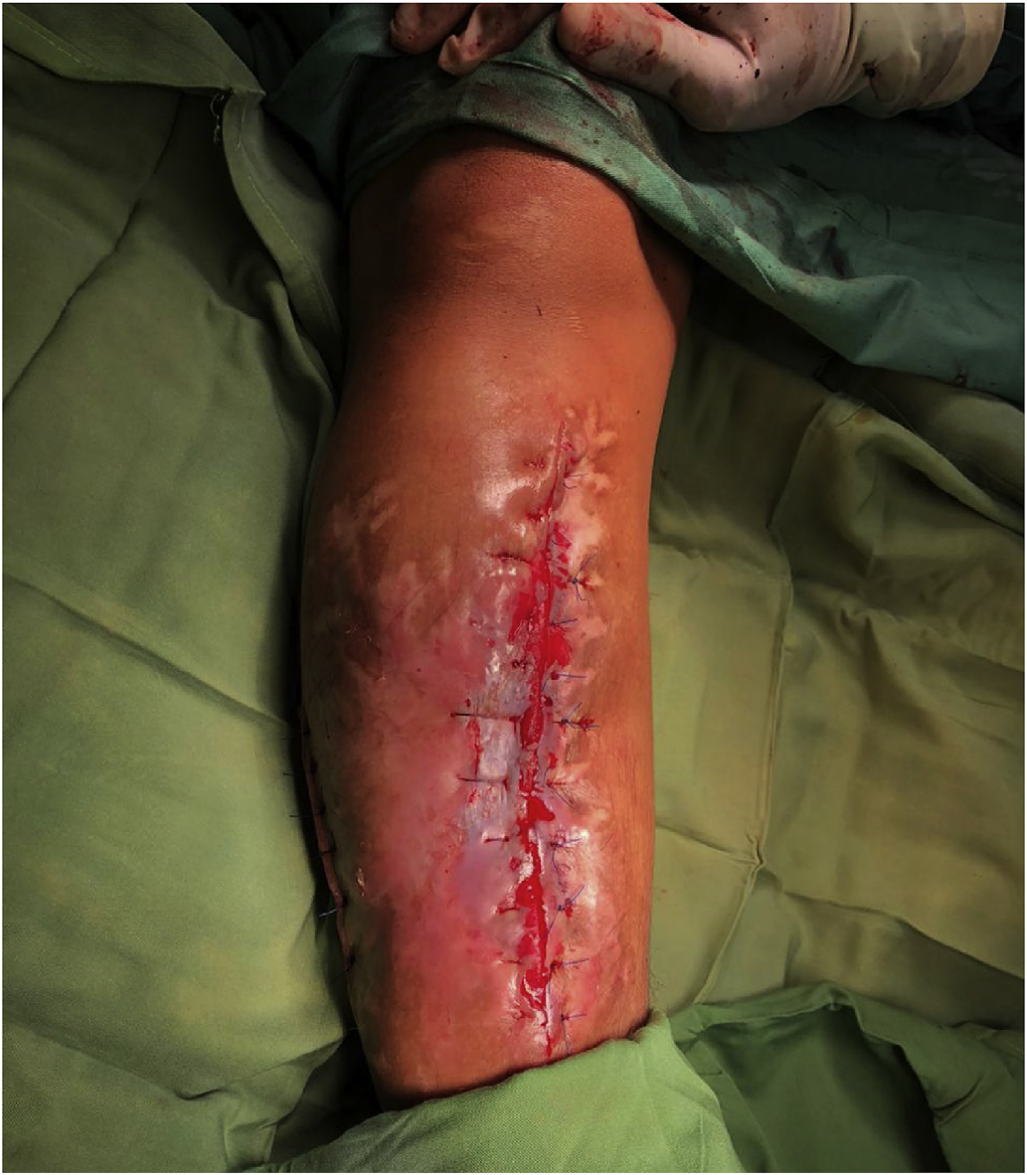
Right leg after debridement and the replacement of internal fixation.

When the patient's body temperature rises to 103.2 °F, treatment proceeded with the intravenous antibiotic cefazolin due to the patient's allergy to penicillin and surgical debridement (Fig. 4) in an effort to control the infection and maintain internal fixation until the fracture fused. As a postoperative outcome, the patient showed satisfactory progress at 6- and 12-month follow-up.

## 3 Discussion

The increased incidence of trauma in recent years has prompted a higher incidence of fractures treated with internal fixation, among which tibia fractures are reported as one of the most common and complicated. Tibia fractures treated by surgery pose a risk of serious, debilitating infections [4], and in orthopedic surgery, IAFF is a frightening complication that can lead to loss of function, delayed healing, and even amputation [6]. In the case reported here, the debridement of the wound was performed prior to referral to our hospital, and internal fixation was performed when the period of infection was considered to have ended.

Over the past few years, several treatments for such fractures have been developed, including non-operative management, minimally invasive treatments, external fixation (ExFix), and open reduction and internal fixation (ORIF) [8,9]. ORIF and ExFix are two methods often reported in the literature. Whereas ORIF can restore the anatomical structure of bone but cannot prevent soft tissue surgery that causes tissue damage and results in longer recovery [10], ExFix allows for an indirect reduction but causes less soft tissue damage. However, several studies have reported that ExFix is associated with high rates of non-union and malunion [11].

A patient's preference of management coverage between external or internal fixation depends upon the patient's desires, needs, and finances, and all surgical procedures should follow the consensus published by the American Academy of Orthopedic Surgeons [12]. Postoperatively, the limb was elevated, and 2–3 weeks later, the suture was removed. On Day 2 postoperatively, brief, non-weight bearing exercises began in bed. The toe-touch weight-bearing of the legs with the aid of two crutches began when the postoperative control X-ray showed the presence of osteotylus and continued 4–6 weeks. Within 3 months, the weight-bearing gradually increased until realizing its full potential [13].

Many concepts of medical care and surgery currently applied to IAFF have been adopted from the prosthetic joint infection (PJI) treatment algorithm [5]. However, after some surgeons questioned the algorithm, it became clear that the guidelines of the Centers for Disease Control and Prevention (CDC) and the guidelines for prosthetic infections were not optimal for treating patients with IAFFs [6].

Accurately estimating the impact of complications related to fractures has been hampered by the lack of clear definitions of complications such as infection or non-union, as well as a lack of consensus and of standard criteria regarding the definition of *IAFF*. By contrast, a clear definition of *PJI* exists [14]. Literature on trauma often mentions the CDC's Guide for Surgical Site Infections, which, according to the CDC's classification, is divided into superficial, deep incisional, and space or organ. Meanwhile, osteomyelitis is classified separately. Because neither the fracture nor the implants are considered, the complexity of the traumatic fracture accompanied by infection is not fully included in those guidelines [5].

Despite clear definitions, different classifications for IAFFs exist. In 1986, Willenegger and Roth classified IAFFs based on time, according to the onset of symptoms, into three groups: early onset (i.e., <2 weeks), delayed onset (i.e., 2–10 weeks), and late onset (i.e., >10 weeks) [6].

In our case, the patient developed a late infection more than 10 weeks after ORIF surgery. Late infection, as in our case, is usually caused by microorganisms with low virulence levels such as *S. epidermidis*. Disruption of the healing a fracture is a clinical symptom that often arises in late infections, marked by the presence of osteomyelitis with sequestrum or involucrum [6]. In such late infections, a compromised healing process often occurs, and although bone healing may have occurred in some cases, severe inflammation is possible, and osteolysis accompanied by osteomyelitis may cause unstable osteosynthesis (Fig. 2). The formation of new periosteal bone around the edge of the infected area produces involucrum, which further distances the infected area. Such changes often require extensive and repeated debridement, which can cause bone defects [5].

Most IAFFs are caused by a group of bacteria that grow in necrotic bone tissue and protect biofilms in foreign materials. Among them, localized bacteria are often metabolically silent, which makes them difficult to culture and identify. Cultures taken from an open wound at the beginning of IAFFs do not always correlate with possible later infection and therefore should be avoided. Likewise, a swab culture during a second repair operation cannot be considered to represent pathogens that cause bone infection and therefore is not indicated, either. When infection is suspected, at least three bone biopsies should be performed in the area around the implant and in areas that appear visibly infected—for example, non-union or necrotic bone tissue [5]. If two separate biopsy cultures have the same microorganisms, then cultures result is considered to be significant. However, in highly virulent species such as *S. aureus* or *E. coli*, one positive biopsy is sufficient to represent the bacterium causing the infection [14].

In our case, the patient developed a late infection, and the goal of treatment was to reduce, if not eradicate, the infection. Eradication would have been the better option, given the difficulty of treating germs and if the quality of soft tissue had been poor; if either of those two criteria had existed, then exchanging management in two stages with debridement and antibiotics for 6 weeks (i.e. intravenously for 2 weeks and orally for 4 weeks) and the installation of external or internal fixation, followed by antibiotics (IV for 1 week and orally for 5 weeks) and re-osteosynthesis, would have been preferred. By contrast, if no such criteria existed, then a single-stage exchange would have been recommended involving the administration of antibiotics for 12 weeks.

In suppressive therapy, debridement is recommended, followed by IV antibiotics for 2 weeks, followed by long-term oral antibiotics until removal of the internal fixation device. The treatment of late infections needs to be planned by using imaging to identify dead bone and sequestrum. During surgery, an orthopedic surgeon should evaluate bone bleeding to assess its viability. A necessary resection can cause a significant loss of stability and require more rigorous reconstruction methods, including bone transport and Masquelet-induced membrane techniques [6].

## 4 Conclusion

Delayed union and chronic osteomyelitis are possible complications of IAFF.

## Funding

No funding or sponsorship.

## Ethical approval

The study is exempt from ethical approval in our institution.

## Author contribution

Muhammad Ardi Munir: Conceptualization, Methodology. Pascal Adventra Tandiabang: Data curation, Writing- Original draft preparation. Prihantono: Visualization, Investigation. Pascal Adventra Tandiabang: Supervision.: Muhammad Ardi Munir: Software, Validation.: Muhammad Ardi Munir: Writing- Reviewing and Editing: Prihantono. All authors read and approved the final manuscript.

## Registration of research studies

None.

## Guarantor

Muhammad Ardi Munir.

## Provenance and peer review

Not commissioned, externally peer reviewed.

## Declaration of competing interest

Nothing to declare.
